# A Novel Multifunctional Nanowire Platform for Highly Efficient Isolation and Analysis of Circulating Tumor-Specific Markers

**DOI:** 10.3389/fchem.2018.00664

**Published:** 2019-01-15

**Authors:** Jiyun Lim, Mihye Choi, HyungJae Lee, Ji-Youn Han, Youngnam Cho

**Affiliations:** ^1^Biomarker Branch, National Cancer Center, Goyang, South Korea; ^2^Department of Cancer Biomedical Science, Graduate School of Cancer Science and Policy, Goyang, South Korea; ^3^Department of Medical Science, Yonsei University College of Medicine, Seoul, South Korea; ^4^Center for Lung Cancer, National Cancer Center, Goyang, South Korea; ^5^Genopsy Inc., Seoul, South Korea

**Keywords:** plasma, exosome, circulating tumor cells, lung cancer, nanowire

## Abstract

Circulating tumor-specific markers are crucial to understand the molecular and cellular processes underlying cancer, and to develop therapeutic strategies for the treatment of the disease in clinical applications. Many approaches to isolate and analyze these markers have been reported. Here, we propose a straightforward method for highly efficient capture and release of exosomes and circulating tumor cells (CTCs) in a single platform with well-ordered three-dimensional (3D) architecture that is constructed using a simple electrochemical method. Conductive polypyrrole nanowires (Ppy NWs) are conjugated with monoclonal antibodies that specifically recognize marker proteins on the surface of exosomes or CTCs. In response to electrical- or glutathione (GSH)-mediated stimulation, the captured exosomes or cells can be finely controlled for retrieval from the NW platform. A surface having nano-topographic structures allows the specific recognition and capture of small-sized exosome-like vesicles (30–100 nm) by promoting topographical interactions, while physically blocking larger vesicles (i.e., microvesicles, 100–1,000 nm). In addition, vertically aligned features greatly improve cell capture efficiency after modification with desired high-binding affinity biomolecules. Notably, exosomes and CTCs can be sequentially isolated from cancer patients' blood samples using a single NW platform via modulating electrochemical and chemical cues, which clearly exhibits great potential for the diagnosis of various cancer types and for downstream analysis due to its facile, effective, and low-cost performance.

## Introduction

Recent advances in the detection and analysis of circulating tumor-derived biomarkers [i.e., circulating tumor cells (CTCs), circulating tumor DNA (ctDNA), or exosomes] have led to a better understanding of the complex molecular, cellular, and genetic processes that govern tumor development and behavior (Wang et al., 2009; Gold et al., 2015; Zhang et al., 2017). Due to their minimally invasive nature, these tumor-related markers afford broad tumor profiling and analysis, and provide sufficient evidence to establish a personalized strategy, which benefits in predicating longitudinal tumor dynamics, tracking the metastatic potential, monitoring in the assessment of response to therapy, and thus help deciding specific treatment plans with improved outcomes (Speicher and Pantel, 2014; Wan et al., 2017).

In particular, since elevated exosome levels are expected in the plasma of cancer patients, their isolation and detection offer novel insights into tumor growth and progression, thereby providing an opportunity for convenient and non-invasive diagnosis of the disease and assessment of therapy response (Melo et al., 2014; Yu et al., 2015). In general, exosomes are small lipid membrane vesicles that contain diverse biomolecules, including intact proteins, DNA, mRNA, and microRNA, which are implicated in the regulation of several cellular functions and in the activation/inhibition of intercellular signaling and communication to adjacent or remote cells (Chiba et al., 2012). Therefore, qualitative and quantitative analysis of exosomal content may provide predictive and prognostic clues in deciding cancer diagnosis and therapeutic approach. Tumor-derived exosomes are commonly 30–100 nm in diameter and are secreted directly from the endolysosomal pathway. On the other hand, extracellular microvesicles (EMVs) are formed by outward buddings that shed from the cell membrane, have heterogeneous shape, and are 100–1000 nm in diameter (Liga et al., 2015; Pedersen et al., 2015). Accordingly, isolation of exosomes with high purity and functional integrity, while preferentially excluding non-exosomal particles (e.g., EMVs), is necessary to acquire key mechanisms implicated in various physiological and pathological processes. Numerous techniques have been developed for the isolation of exosomes, including ultracentrifugation, density gradient centrifugation, size exclusion, precipitation, and immunoisolation (Tauro et al., 2012; Baranyai et al., 2015; Rider et al., 2016). Conventional differential centrifugation involves several centrifugation, filtration, and ultracentrifugation steps to directly accumulate membrane-bound vesicles. However, along with exosomes, this technique extracts protein aggregates and extracellular vesicles of various sizes. In addition, it requires laborious and time-consuming purification processes, resulting in low exosome efficiency and purity. Immunoisolation assays have been developed for rapid exosome isolation based on exosome surface-specific proteins or antibodies coupled with magnetic beads or microfluidic devices (Jørgensen et al., 2013; Kanwar et al., 2014; Zhao and He, 2014).

**Figure 1:**
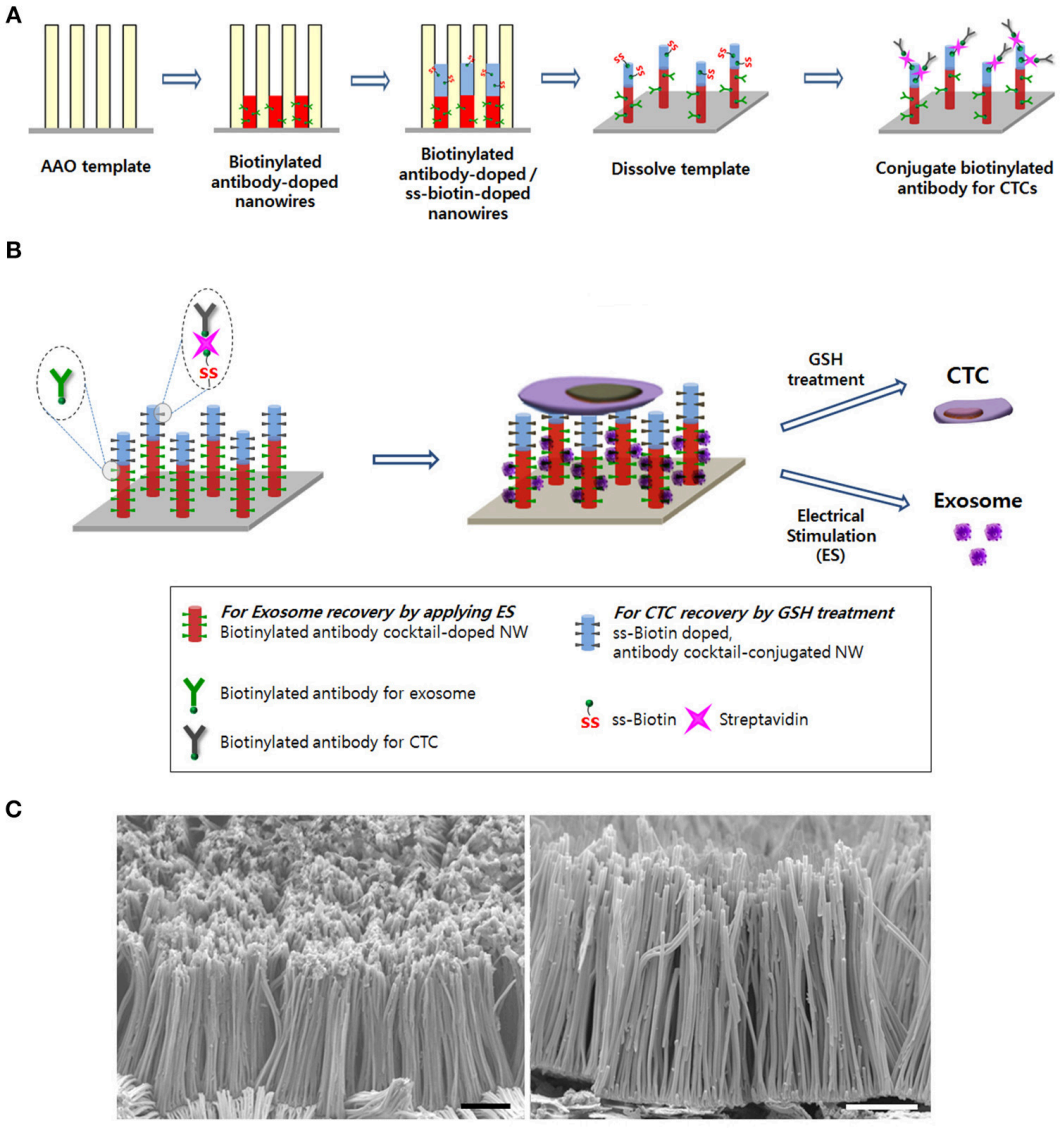
**(A)** Schematic of the process to synthesize the Ppy nanowire (NW) platform conjugated with simultaneously exosome-specific antibodies and CTC-targeting antibodies. **(B)** Schematic illustration of exosome and circulating tumor cells (CTCs) recovery from the same NW array (200 nm in diameter and 14 μm in length). This newly introduced platform possesses unique features by means of (i) doping exosome-specific biotinylated antibodies (i.e., anti-CD9, anti-CD81, and anti-CD63) to the bottom of the NW that could greatly enhance exosome isolation via the application of electrical stimulation (ES) and (ii) doping disulfide (SS)-biotin to the top of the NW that is further conjugated with CTC-targeting antibodies (i.e., anti-EpCAM, anti-EGFR, anti-vimentin, anti-N-cadherin, and anti-Trop2), which consequently releases the captured CTCs from the NW platform via GSH treatment. **(C)** Scanning electron microscopy images of the nanowire platform (scale bars = 5 μm).

In this study, we present a technically simple and efficient approach for the isolation and molecular analysis of exosomes from diverse biological samples using a free-standing three-dimensional (3D) polypyrrole nanowires (Ppy NWs) platform. Surfaces with homogeneous nanoscale topography labeled with antibodies targeting exosome surface markers offer enhanced performance in separation of pure exosomes. Indeed, nanoscale interfaces between NWs are advantageous for trapping exclusively small membranous exosomes, while simultaneously excluding EMVs and cell debris, thereby increasing the efficiency of exosome-like lipid vesicles isolation. Furthermore, vertically aligned NW arrays simultaneously serve as a versatile multifunctional platform to improve the extraction of CTCs. This unique NW interface is an ideal candidate for companion extraction of exosomes and CTCs using the same platform by electrical stimulation (ES) or glutathione (GSH) treatment, and for clinical applications in cancer diagnostics and therapy (Figure 1).

## Experimental

### Materials

Pyrrole, poly(sodium 4-styrenesulfonate) (PSS), N-(3-dimethylaminopropyl)-N'-ethylcarbodiimide hydrochloride (EDC), N-hydroxysuccinimide (NHS), streptavidin, and sodium hydroxide (NaOH) were obtained from Sigma Aldrich (St. Louis, MO, USA). Anodized aluminum oxide (AAO) membrane filter (pore diameter, 200 nm) was purchased from Whatman (Pittsburgh, PA, USA). Silver paste (P-100 type) was obtained from CANS. NHS-SS-biotin was purchased from CovaChem (Loves Park, Illinois, USA). Biotinylated anti-EpCAM, anti-EGFR, anti-vimentin, anti-N-cadherin, and anti-Trop2 were obtained from R&D system (Minneapolis, MN, USA). Biotinylated anti-CD63 and anti-CD81 were purchased from AnCell (Oak Park, Minnesota, USA). Biotinylated anti-CD9 was obtained from Abcam (Cambridge, UK).

### Fabrication and Characterization of Free-Standing Ppy NW Platform

All electrochemical experiments were carried out using a potentiostat/galvanostat (BioLogic SP-50) with platinum wire, Ag/AgCl (3 M NaCl type), and an AAO disc on indium tin oxide (ITO) glass as counter, reference, and working electrodes, respectively. To prepare Ppy NW arrays with two different functions, Ppy NWs in the pores of the AAO template were electrochemically deposited in two aqueous solution sets of (i) a mixture of 0.01 M pyrrole and 0.01 M PSS containing anti-CD9, anti-CD81, and anti-CD63 (10 μg/mL in phosphate-buffered saline; PBS) to obtain a final antibody concentration of 0.18 μg/mL and (ii) a mixture of 0.01 M pyrrole and 0.01 M PSS containing 1 mg/mL NHS-SS-biotin by cyclic voltammetry. After performing electropolymerization, the resulting AAO templates were immediately rinsed with ultrapure water 3 times and allowed to dry in the air. Then, the AAO templates were fixed onto the ITO glass with silver paste and incubated overnight at 63°C. Finally, the AAO template was incubated with 2 M NaOH at room temperature to obtain free-standing Ppy NWs. The resulting Ppy NWs were further reacted with 5 mL of 30 mM EDC/6 mM NHS solution for 1 h to activate the carboxylic groups of SS-biotin. After 3 washes with ultrapure water, the resulting Ppy NWs were incubated with 5 mL of streptavidin (10 mg/mL in dH_2_O) for 1 h and precipitated with distilled water 3 times. Subsequently, 5 mL of biotinylated anti-EpCAM, anti-EGFR, anti-vimentin, anti-N-cadherin, and anti-Trop2 (10 μg/mL in PBS) were added for conjugation to streptavidin-labeled SS-biotin-Ppy NWs to achieve a final antibody concentration of 0.22 μg/mL. The suspension was reacted at 4°C overnight. The microstructures of the dually functionalized Ppy NWs array were observed using a field-emission scanning electron microscope (JSM-7800F, JEOL) with an accelerating voltage of 12 kV. To quantify the number of antibodies per free-standing Ppy NW platform, the Ppy NW platform conjugated with exosome-specific antibodies or CTC-targeting antibodies was incubated with horseradish peroxidase-labeled (HRP) anti-mouse IgG for 1 h. A 3% solution of bovine serum albumin (BSA) was used to block non-specific binding. The Ppy NW platform was washed several times to remove any unbound IgG. HRP bound to the Ppy NW was reacted with 3,3′,5,5′-tetramethyl benzidine to generate a colored product and compared with an HRP anti-mouse IgG standard curve ranging from 2 × 10^−5^ to 2 × 10^−9^ g/mL. The results were read using a spectrophotometer at 450 nm.

### Cell Culture and Preparation of Exosome-Concentrated Culture Medium (CCM)

Four different types of cancer cell lines (i.e., MDA-MB-231 and MCF7 breast cancer cells, HCT116 colon cancer cells, NCI-H358, and NCI-H460 lung cancer cells) were cultured in Roswell Park Memorial Institute (RPMI)-1640 medium (Invitrogen, Carlsbad, CA) containing 10% fetal bovine serum (FBS) and 1% penicillin-streptomycin at 37°C in a 5% CO_2_ atmosphere. The cells (~2 × 10^9^ cells) were washed twice with DPBS, followed by replacement of serum-free RPMI medium. The cells were incubated for additional 2 days in serum-free RPMI medium before exosome harvesting. Intact cells and cell debris were removed by centrifugation at 300 × *g* for 10 min and 2,000 × *g* for 20 min, respectively. Then, CCM was collected by filtration through sterile 0.22-μm (pore-size) syringe filter (Merck Millipore, USA).

### Isolation of Exosomes by Ppy NWs Array

Captured exosomes were released from the Ppy NWs array by applying electrical stimulation. To isolate exosomes, the antibody-labeled Ppy NWs array was incubated in CCM or the plasma of healthy donors and cancer patients for 40 min at room temperature with gentle shaking (500 rpm) to induce attachment of exosomes to the nanowires. Next, to induce ES-mediated exosome release, the Ppy NWs array with the captured exosomes was assembled in a plate material-evaluating cell with three electrodes consisting of three electrodes, a reference (Ag/AgCl), counter (Pt), and working electrode (PPy NWs), and electrical stimulation was applied at +0.5, 0, −0.5, −1.0, and −1.5 V for 3 min to elute the captured exosomes from the NWs to DPBS. To compare the performance, the exosomes were also isolated and purified using ExoQuick (EXOQ5TM-1, System Biosciences, Palo Alto, CA, USA) and the Invitrogen Total Exosome Isolation Kit (4484451, Thermo Fisher Scientific, Massachusetts, Waltham, USA) according to the manufacturer's protocols. Briefly, the reagents were added to CCM or plasma of healthy donors and cancer patients to collect exosomes, and the mixture was vortexed and centrifuged at 4°C as described in the manufacturers' instructions. The pellet containing exosomes was resuspended in DPBS or ultrapure water. Next, the concentration and size distribution of the exosomes isolated with the Ppy NWs array or conventional extraction kit were evaluated using the nanoparticle tracking analysis (NTA) software (NanoSight NS300, Malvern Instruments, Malvern, UK) and the Malvern Zetasizer Nano-Z (Malvern Instruments, Malvern, UK). All measurements were carried out in triplicate to obtain consistent results.

### Western Blotting

Exosomes isolated with the Ppy NWs array were lysed in M-PER reagent (Thermo Fisher Scientific, Massachusetts, Waltham, USA). For equal volume loading measurements, protein concentration was measured using the bicinchoninic acid (BCA) assay kit (Thermo Scientific, Waltham, MA). Protein samples (20 μg) were separated on a 10% sodium dodecyl sulfate polyacrylamide gel and transferred onto polyvinylidene difluoride (PVDF) membranes (0.45 μm, Millipore). The membranes were blocked in 3% skim milk for 1 h at room temperature and incubated overnight with primary rabbit anti-HSP70 (1:1000), rabbit anti-glyceraldehyde 3-phosphate dehydrogenase (GAPDH) (1:1000), rabbit anti-CD9 (1:1000), and rabbit anti-CD81 (1:1000). Following incubation, the membranes were incubated with the appropriate secondary antibody (goat anti-mouse IgG [1:3000] or goat anti-rabbit IgG [1:3000]) for 1 h. After 3 washes in TBS-T, signals were visualized using the SuperSignal® West Pico Chemiluminescent Substrate reagent (34077, Thermo Scientific).

### Cell Capture and Release From Artificial Blood Samples

Ppy NW platforms doped with (i) exosome-specific biotinylated antibodies (i.e., anti-CD9, anti-CD81, and anti-CD63) to the bottom of the NW and (ii) disulfide (SS)-biotin to the top of the NW, which was further conjugated with CTC-targeting antibodies (i.e., anti-EpCAM, anti-EGFR, anti-vimentin, anti-N-cadherin, and anti-Trop2) were placed in 12-well culture plates. Varying amounts (3–100 cells/mL) of cancer cells (i.e., MCF7, HCT116, H1975, and H460) were spiked into 1 mL of blood from a healthy donor. Next, the cell suspensions were seeded on Ppy NW platforms for 30 min to enhance attachment of the target cells to the Ppy NW platforms, and the surface was washed with PBS 3 times. The Ppy NW platform was incubated with the captured cells in 50 mM GSH solutions for 0, 10, and 30 min with gentle shaking at 500 rpm. The GSH-mediated released cells were identified as CTCs by dual staining with EpCAM (green) and DAPI (blue) and by observing the morphology of the cancer cells. Cells subjected to dual staining [CD45+ (red) and DAPI+ (blue)] were classified as non-specific hematological cells such as white blood cells (WBCs). The released cells were washed with 1× PBS, resuspended in 500 μL of 4% paraformaldehyde (PFA) solution, and transferred to a glass slide. The isolated cells were incubated at 37°C overnight for immunofluorescence (IF). The cells were incubated with anti-EpCAM, anti-Vimentin, and anti-CD45 diluted in 5% BSA/PBS for 90 min. After 3 washes with DPBS, the cells were incubated with Alexa Fluor 488-, 568-, and 647-conjugated (Invitrogen) secondary antibody (green signal for EpCAM, red signal for vimentin, and purple signal for CD45, respectively) diluted in 5% BSA/PBS for 40 min. The secondary antibodies were diluted at the same ratio as the primary antibody. Finally, the cells were stained with 6-diamidino-2-phenylindole (DAPI) (Hoechst 33258, 1:10,000 dilutions in 1X DPBS) and observed with a fluorescence microscope (Axio Imager M2, Carl Zeiss, Oberkochen, Germany).

### Isolation of Circulating Tumor Cells From Patient Blood

For clinical application, no humans were part of this study, and all samples were acquired from the National Cancer Center Hospital (Goyang, Korea) according to procedures approved by the National Cancer Center (NCC) Institutional Review Board guidelines (NCC2016-0208). Whole blood collected in EDTA Vacutainer tubes (Becton and Dickinson) was diluted by 5-fold with 0.1% PBS/BSA. The diluted blood was incubated onto the Ppy NW platform conjugated with exosome-specific antibodies and CTC-targeting antibodies by shaking for 30 min at room temperature, and the resulting surface was washed several times with PBS. We next incubated the Ppy NWs with captured cells in 50 mM GSH solutions for 30 min with gentle shaking at 500 rpm to isolate CTCs from the platform. The released cells were washed with 1 × PBS, resuspended in 500 μL of 4% PFA solution, and transferred to a glass slide. The isolated cells were incubated at 37°C overnight for immunofluorescence (IF).

### Transmission Electron Microscopy (TEM) Analysis of Exosomes

Exosomes freshly isolated from cells or plasma of cancer patients were resuspended in cold DPBS. Exosome samples were prepared for TEM analysis using the exosome-TEM-easy kit (101Bio, Palo Alto, CA, USA) according to the manufacturer's instructions. Briefly, re-suspended exosomes were mounted on formvar/carbon-coated EM 400 mesh grids and incubated for 5 min. The grids were rinsed twice with wash buffer and deposited in the EM solution for 2 min. After washing and dehydration steps, exosomes were observed by TEM (JEM-F200, JEOL) with an accelerating voltage of 300 kV.

### Extraction and Quantitative Reverse Transcription Polymerase Chain Reaction (qRT-PCR) Analysis of Exosomal RNA

Total RNA was extracted from the exosomes isolated with the Ppy NWs array using TRI REAGENT (Molecular Research Center, Cincinnati, OH, USA). After treatment with TRIzol and chloroform (Merck, Darmstadt, Germany), exosome samples were centrifuged at 12,000 × *g* for 15 min at 4°C to separate the mixture into aqueous and organic phases. RNA was precipitated from the supernatant in presence of isopropanol. After precipitation, miR21-specific complementary DNA (cDNA) was synthesized from 10 ng of total RNA using the TaqMan MicroRNA reverse transcription Kit (Applied Biosystems, Foster city, CA, USA) and miR21-specific primers according to the manufacturer's protocol. Primers and probe pre-designed for RT reaction or qRT-PCR (Applied Biosystems) were used. qPCR was performed in 384-well reaction plates using an LC480 real-time PCR system (Roche, Basel, Switzerland).

## Results and Discussions

### Capture and Electric-Field Responsive Controlled Release of Exosomes

**Figure 2:**
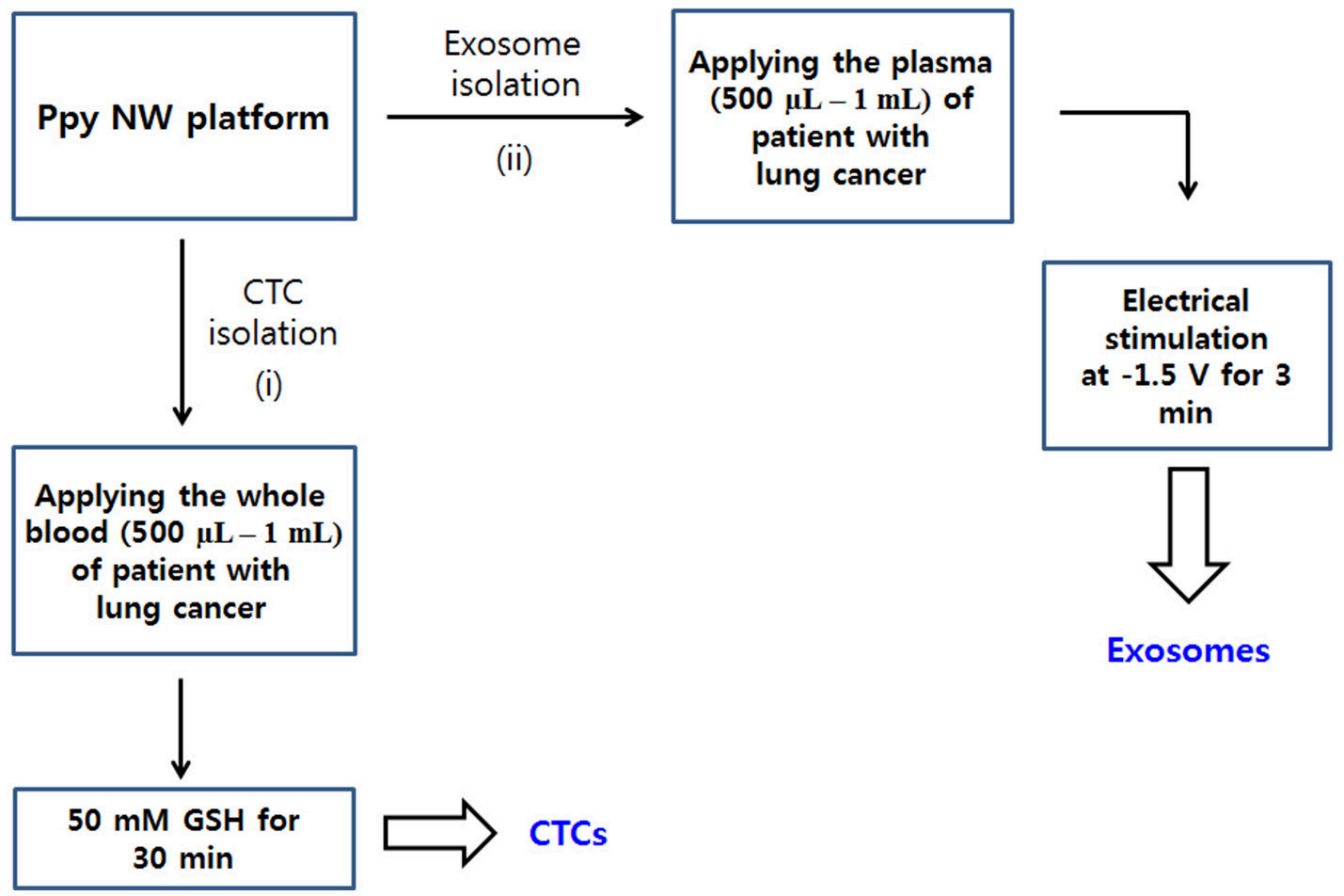
Workflow for isolating CTCs (i) and exosomes (ii) from body fluid samples of cancer patients. Ppy NWs conjugated with simultaneously exosome-specific antibodies and CTC-targeting antibodies allow for continuous extraction of exosomes and CTCs from a single platform by applying electrical stimulation (ES) and GSH treatment.

**Figure 3:**
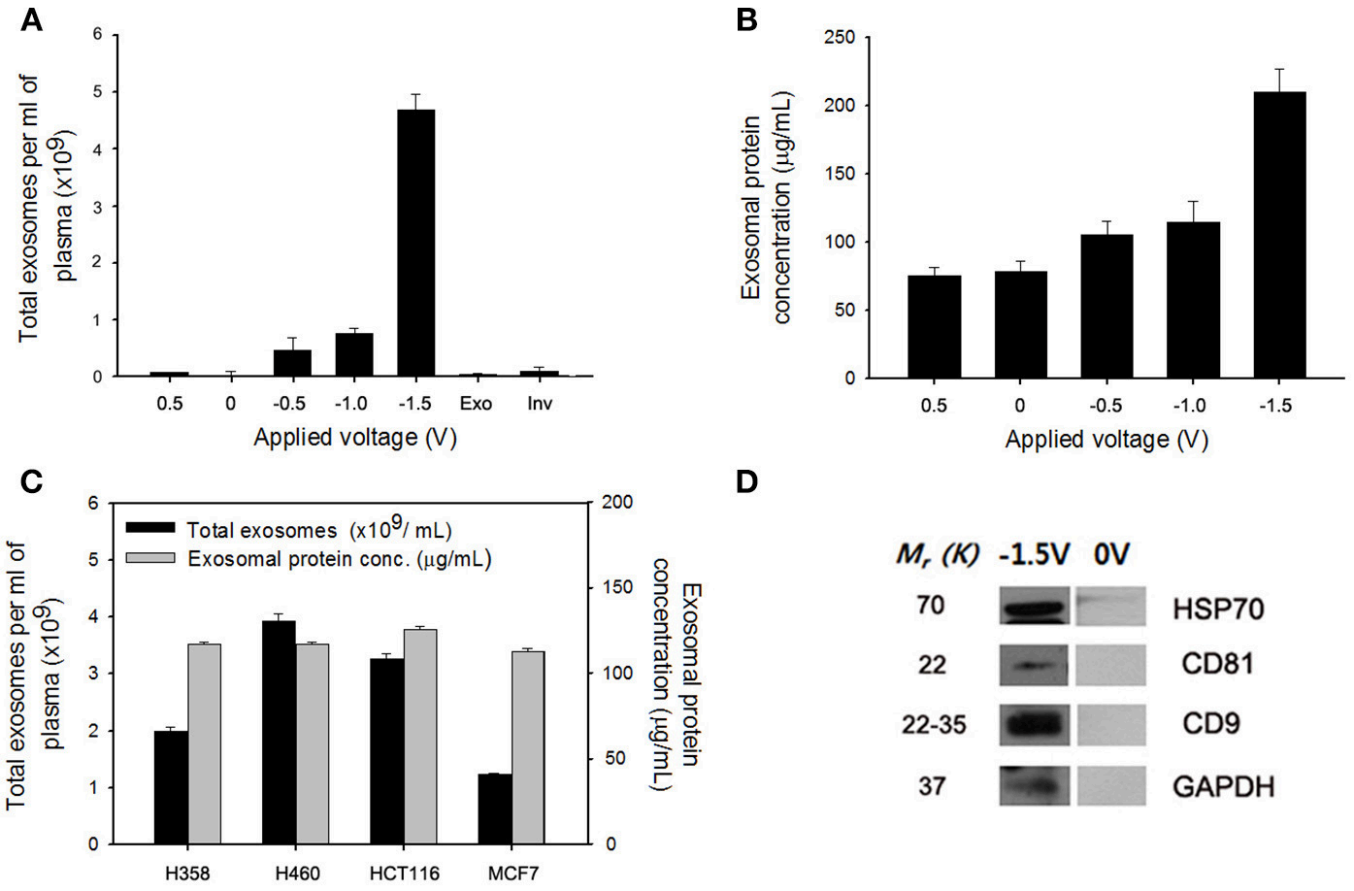
**(A)** Electric field-mediated controlled isolation of exosomes from the culture medium of MDA-MB-231 breast cancer cells using the nanowires (NWs) platform in response to various electrical potentials. **(B)** Quantification of the exosomal proteins isolated from the NW platform at various potentials. **(C)** Verification of the exosome yield and exosomal proteins level from four different cancer cell lines (i.e., NCI-H358 and NCI-H460 lung cancer cells, MCF7 breast cancer cells, and HCT116 colon cancer cells) upon application of −1.5 V. **(D)** Western blotting of exosomes released at −1.5 V to evaluate the expression level of exosomal proteins isolated from the culture medium of MCF7 cells. Data are shown as the mean ± standard deviation (*n* = 5).

As shown in Figure 1, specific targeting ligands can be easily incorporated to the NWs by electrochemical deposition, in which biotinylated antibodies are doped to the bottom of the nanowire for exosome extraction, whereas SS-biotin molecules are incorporated to the top of the nanowire, followed by efficiently accommodating CTCs-specific antibodies. The process of preparing a vertically aligned Ppy NWs array for the recovery of exosomes and CTCs is illustrated in Figures 1A, 2. As evidenced in a previous study, Ppy undergoes reversible polymeric volume change in response to applied electrical field. Ppy film expansion in the oxidized state is preferable for entrapment of large amounts of biomolecules into the polymeric backbone (Otero and Martinez, 2013; Saitou, 2013; Jeon et al., 2014). On the other hand, reduced Ppy backbone results in distinct shrinkage, thereby expelling the attached molecules. First, Ppy nanowires (200-nm diameter pores) carrying two different functionalities were successfully synthesized by potentiostatic electrodeposition on an AAO template. Two mixtures consisting of (i) pyrrole monomers and exosome-specific biotinylated antibodies (i.e., anti-CD9, anti-CD81, and anti-CD63) and (ii) pyrrole monomers and SS-biotin were used to accomplish electropolymerization into the AAO nanopores. Subsequently, upon immediate removal of the AAO template, SS-biotin molecules were further decorated with CTCs-specific biotinylated antibody cocktail (i.e., anti-EpCAM, anti-EGFR, anti-vimentin, anti-N-cadherin, and anti-Trop2) through streptavidin-biotin recognition. SEM images clearly show a well-defined morphology of the NWs with length of approximately 14 nm and diameter of 200 nm (Figure 1B). The length of NWs can be controlled by finely adjusting the polymerization conditions. Next, as a “proof-of-concept,” electric field-mediated exosomal isolation was tested using the CCM of MDA-MB-231 breast cancer cells in order to validate the electrochemical behavior of the proposed platform and compare the results with those of commercial kits (Figure 3A). Vertically grown free-standing NWs offer large surface-to-volume area that provides high loading capacity of the target antibodies, ultimately allowing high-density capture of exosomes compared to that of conventional methods. Taking advantage of the intrinsic redox reaction of electroactive Ppy, the NWs platform with captured exosomes can swell and contract in response to various electric potentials, which ultimately induces the release of exosomes from the platform. Exosome capture efficiency was investigated by application of electrical potentials. As expected, immediately following exposure to −1.5 V, high-yield and -purity exosomes were readily and efficiently isolated from CCM with an average NTA value of 4.5 × 10^9^ exosomes/mL, which is attributed to the physical compression of the reduced state of Ppy backbone, ultimately facilitating the release of conjugated antibodies and attached exosomes (Figure 3A). Moreover, exosomes isolated using the Exoquick and Invitrogen kits provided an approximate NTA value of 0.2 × 10^9^ and 0.31 × 10^9^ exosomes/mL, respectively, which resembles the concentration of exosomes isolated at positive potential and is possibly associated with naturally occurring release. When a voltage of −1.5 V was applied to the NW platform, the yield of the proposed platform was approximately 14-fold higher than that of the two commercial methods. The amount of exosomal proteins at various electrical potentials was also evaluated (Figure 3B). As expected, the levels of exosomal proteins were markedly elevated when isolation occurred after exposure to −1.5 V for 3 min. Indeed, the greater the amount of exosomes recovered, the greater the amount of exosome proteins detected. The performance of the NW platform in terms of exosome yield and exosomal proteins levels was further validated using four different cancer cell lines (i.e., NCI-H358 and NCI-H460 lung cancer cells, MCF7 breast cancer cells, and HCT116 colon cancer cells; Figure 3C) upon application of −1.5 V. The newly introduced NW platform provides exosomes and exosomal proteins at high yield regardless of the cell lines used, indicating that the well-defined NW structure makes them ideal for highly efficient exosome isolation with rapid reaction time (i.e., < 1 h). Exosomes isolated from the culture medium of MCF7 cells were analyzed to assess the presence of common exosome-associated proteins on their membrane surfaces (Figure 3D). The yield of the NW platform was approximately 220 μg of proteins from ~5 × 10^6^ cells. Western blot analysis showed the presence of exosome markers such as CD9, CD81, HSP70, and GAPDH. These unique 3D NW arrays possess distinct advantages in exosome-capture and release performance in response to the applied electrical potentials.

### Capture and GSH-Responsive Controlled Release of CTCs From Artificial Blood Samples

**Figure 4:**
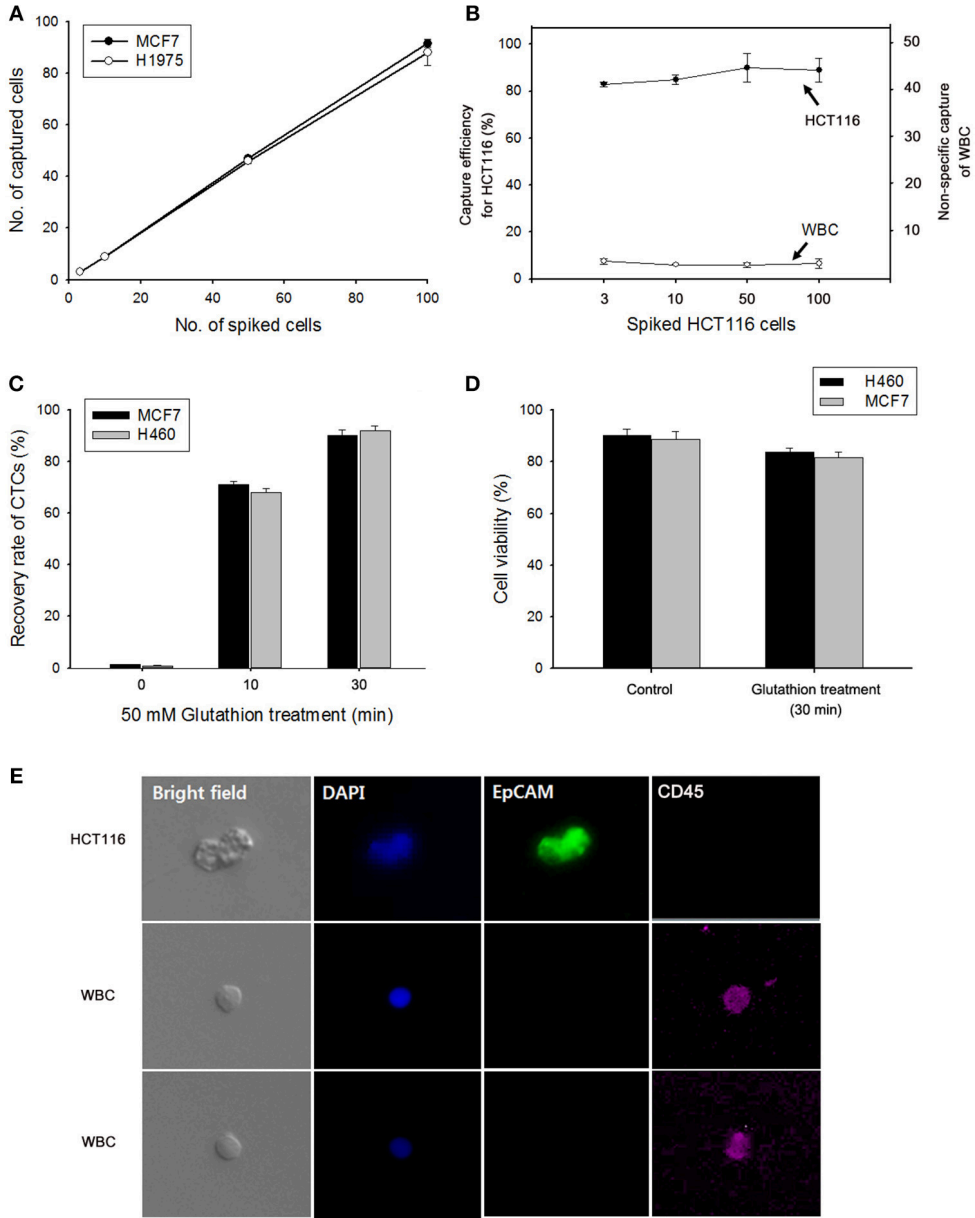
**(A)** Evaluation of cell capture efficiency of the nanowire (NW) platform by spiking MCF7 and H1975 cells into 1 mL of human blood. **(B)** Capture efficiency of HCT116 cells and nonspecifically bound WBCs under different numbers of spiked cancer cells. **(C)** GSH-mediated release of the captured MCF7 and H460 cells from the NW platform at various time points. **(D)** Assessment of the viability of H460 and MCF7 cells detached upon GSH treatment. **(E)** Fluorescent micrographs of GSH-triggered cell release from artificial blood samples using a three-color immunocytochemistry method. MCF7 cells were distinctly stained with anti-EpCAM (green) and DAPI (blue), but negatively stained with anti-CD45 (red). Data are shown as mean ± standard deviation (*n* = 5).

The effectiveness of the NW platform in CTC isolation was investigated in Figure 4. To assess the efficiency of the platform, varying numbers (3–100) of cancer cells (i.e., MCF7 breast cancer cells, HCT116 colon carcinoma cells, and H1975 and H460 lung cancer cells) were spiked into 1 mL of healthy donor's blood in accordance with the NCC Institutional Review Board guidelines. As shown in Figure 4A, excellent CTC capture performance was achieved, with an overall capture efficiency of ~ 80% in all cases. Similar to the exosome results, the proposed NW platform is well-suited for promoting enhanced cell adhesion, which greatly enhances the recovery yield of cancer cells. Meanwhile, the number of WBCs captured non-specifically on the NW platform was very low (Figure 4B). Most WBCs non-specifically immobilized likely detached from the Ppy NW platform during GSH treatment for 30 min. The majority of the adherent CTCs was easily detached from the NWs in response to GSH stimulus, because the disulfide bonds in SS-biotin molecules doped inside the Ppy NWs were broken into thiol groups by GSH (Figure 4C) (Li et al., 2008; Kim et al., 2010; Cheng et al., 2011). Indeed, upon exposure to 50 mM GSH, 90% of the captured cells were released from the NW platform within 30 min, while retaining high cell viability even upon GSH treatment (Figure 4D). Cancer cells were also tested by staining with cell-specific markers such as anti-EpCAM, DAPI, and leukocyte-specific anti-CD45. MCF7 cells captured and consecutively released in GSH-triggered systems exhibited distinct positive staining for EpCAM and DAPI, and negative staining for anti-CD45 with normal cell morphology, indicating that these cells maintain their epithelial features (Figure 4E).

### Performance Evaluation of the NW Platform Using the Plasma of Healthy Donors and Patients With Lung Cancer

**Figure 5:**
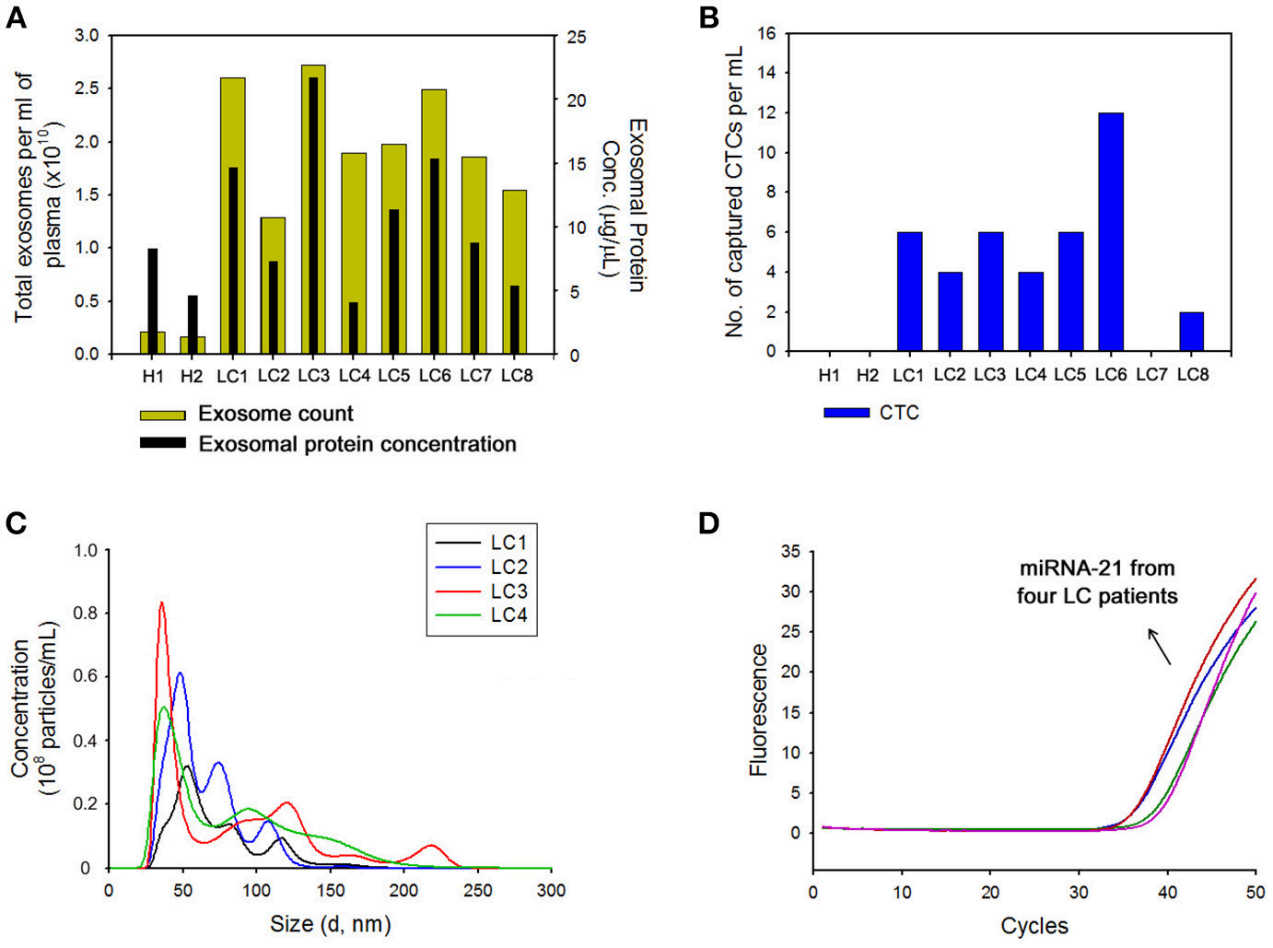
**(A)** Nanoparticle tracking analysis results showing the number of exosomes and total proteins in exosomes collected from the plasma of healthy donors (H) and patients with lung cancer (LC) using the nanowire platform. **(B)** Evaluation of circulating tumor cells (CTCs) capture efficiency from the plasma of healthy donors (H) and patients with lung cancer (LC) using nanowire platform. **(C)** The size distribution of exosomes isolated with the nanowire platform from the plasma of 4 LC patients. **(D)** Reverse transcription-polymerase chain reaction result showing the expression levels of miR-21 in exosomes isolated from the plasma of LC patients with the nanowire platform.

The clinical feasibility of the NW platform was further validated by sequentially extracting exosomes from plasma, followed by CTC from whole blood of lung cancer patients. The NW array with desired multifunctional properties endows a versatile and practical dual extraction strategy with the purpose of high-purity extraction of both exosomes and CTCs. As shown in Figures 5A,B, a high density of exosomes was isolated from 1 mL of plasma of lung cancer patients after exposure to −1.5 V for 3 min. Consistent with the results reported above, total exosome and exosomal protein levels in cancer patients significantly increased compared to that of healthy donors. Besides exosomes, NW arrays offer great advantages in enriching and extracting CTCs. Collectively, 8 blood samples from lung cancer patients and 2 healthy controls were examined. A considerable number of CTCs was identified in 7 out of the 8 cancer patients, whereas no identifiable CTCs were detected in samples from healthy donors. On the other hand, most of the exosomes isolated with the NW platform showed uniform size distribution in the range of 30–150 nm (Figure 5C). Moreover, plasma-derived exosomal RNAs were amplified by RT-PCR to examine the expression level of miR-21 in exosomal RNAs. Figure 5D shows miR-21 positive expression in exosomes isolated from the plasma of four lung cancer patients, which agrees with previous studies reporting that miR-21 is overexpressed in lung cancer(Markou et al., 2016; Xue et al., 2016).

**Figure 6:**
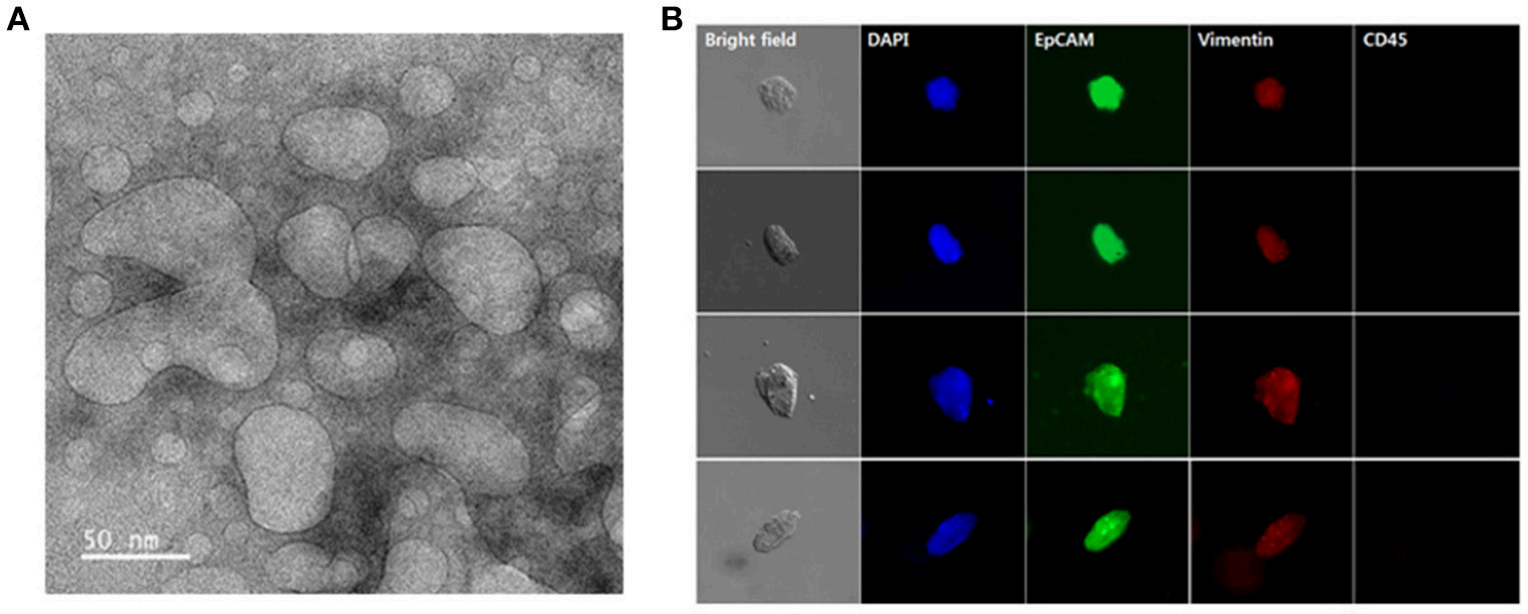
Exosomes and circulating tumor cells (CTCs) were sequentially isolated from the plasma and whole blood of lung cancer patients using the same nanowire platform. **(A)** Transmission electron microscopy image displaying the presence of exosomes isolated from 1 mL of plasma from lung cancer patients (scale bar, 50 nm). **(B)** Representative immunocytochemistry images of CTCs captured from 1 mL of whole blood from lung cancer patients. Cells were stained with DAPI (nucleus; blue), CD45 (hematopoietic; red), EpCAM (epithelial; green), and vimentin (mesenchymal; red).

The presence of exosomes in our samples was further confirmed by TEM. Separation with the NW platform clearly revealed abundance of small vesicles, particularly in a size range of 30–100 nm, confirming that the NW platform-based method ensures isolation of homogeneous exosome population with high yield and purity (Figure 6A). In addition, cells isolated from lung cancer patient samples were further characterized by morphological and immunocytochemistry (ICC) analysis. Conventional labeling protocols were employed to define CTCs based on specific expression of epithelial origin markers (i.e., DAPI+/EpCAM+/CD45–), discriminating from leukocytes that were classically defined as DAPI+/EpCAM–/CD45+ (Figure 6B). Overall, using cancer-specific binding agents, sequential isolation of CTCs and exosomes using the same NW platform with GSH-mediated and electric field-triggered stimulation can be achieved. Indeed, vertically aligned, nanoscale topographic features have been recognized as a versatile and efficient way to enhance the recovery yield of circulating cancer-related markers in a short time (~1 h).

## Conclusion

In summary, a multifunctional NW array conjugated with target specific antibodies is used for sequential isolation of exosomes and CTCs from the plasma and whole blood of lung cancer patients in a single platform. Vertically aligned, three-dimensional morphology of nanowires greatly improves exosome and CTC recovery in < 1 h, by substantially facilitating nanoscale topographical interactions. The NW platform may enable simultaneous isolation and detection of circulating cancer-specific markers with simple preparation and excellent clinical performance, which could greatly contribute to cancer screening and diagnosis.

## Author Contributions

JL, MC, and HL performed the experimental work. J-YH and YC contributed to the analysis and representation of data. YC wrote the manuscript.

### Conflict of Interest Statement

YC was employed by company Genopsy and the National Cancer Center. The remaining authors declare that the research was conducted in the absence of any commercial or financial relationships that could be construed as a potential conflict of interest.
